# Clinical guidelines for the recognition of melanoma of the foot and nail unit

**DOI:** 10.1186/1757-1146-3-25

**Published:** 2010-11-01

**Authors:** Ivan R Bristow, David AR de Berker, Katharine M Acland, Richard J Turner, Jonathan Bowling

**Affiliations:** 1 School of Health Sciences, University of Southampton, SO17 1BJ, UK; 2 Bristol Dermatology Centre, Bristol Royal Infirmary, Bristol, BS2 8HW, UK; 3 St Johns Institute of Dermatology, St Thomas' Hospital, London, SE1 7EH, UK; 4 Department of Dermatology, Oxford Radcliffe Hospital, Oxford, OX3 7LJ, UK

## Abstract

Malignant melanoma is a life threatening skin tumour which may arise on the foot. The prognosis for the condition is good when lesions are diagnosed and treated early. However, lesions arising on the soles and within the nail unit can be difficult to recognise leading to delays in diagnosis. These guidelines have been drafted to alert health care practitioners to the early signs of the disease so an early diagnosis can be sought.

## Overview and scope of the guidelines

Melanoma is a life threatening but potentially treatable form of cancer if diagnosed and managed at an early stage. Guidelines have been published to assist healthcare workers in the recognition of malignant melanoma of the skin [1]. However, early melanoma arising on the foot, particularly within the nail unit and on the plantar surface, can be difficult to recognise. Consequently, this can lead to delays in diagnosis. Melanoma arising on the foot carries a particularly poor prognosis when compared to melanoma arising at other body sites [2-4]. As there are no consistent features of an early melanoma, these guidelines have been drafted to alert health care workers to the signs which may suggest melanoma and therefore warrant a specialist referral. A melanoma recognised and diagnosed at an early stage can dramatically increase a patient's chances of survival.

This guide has been produced as a reference for health care professionals who may be confronted with pigmented and amelanotic lesions on the foot. It has been split into two sections-melanoma on the skin of the foot and melanoma in the nail. The paper is designed to act as a guide in deciding whether a presenting lesion should be referred on. It is not designed to be a diagnostic tool-confirmation of diagnosis can only be secured though appropriate biopsy, histological examination and specialist interpretation. Furthermore, it is appreciated that melanoma is not the only malignant skin tumour arising on the foot. However, these guidelines should alert practitioners to any skin lesions of the foot exhibiting unusual features. If there is any doubt, a second opinion should be sought. At a local level, foot clinics may wish to establish links with their local dermatology and oncology services to facilitate rapid referral pathways.

## What is a melanoma and how common is it?

A melanoma is a malignant tumour (cancer) arising from the pigment producing cell of the skin, the melanocyte. The number of cases of malignant melanoma worldwide is increasing faster than any other form of cancer amongst Caucasians [5]. When compared to other forms of skin cancer, the disease is relatively uncommon [6]. However in the UK, like much of the world, the incidence of cutaneous melanoma continues to rise accounting for the majority of skin cancer deaths. It has been calculated that the lifetime risk for an individual developing the disease is 1:120 for men and 1:95 for women [1]. Currently there are around 8500 new cases annually in the UK with around 1800 melanoma related deaths [7]. Cutaneous melanoma can develop on any skin and mucosal surface. The lower limb is the location of around 30% of all primary cutaneous melanomas, with women are more highly represented in this group, and foot and ankle lesions representing around 3-15% of all cutaneous melanomas [8].

## Who is likely to develop melanoma?

There is a relationship between ultra-violet (UV) exposure and the development of melanoma on sun exposed sites. Data has demonstrated that in particular that irregular and intense exposure to sunlight significantly increases the risk of melanoma [9]. However, the relevance of UV light on non-exposed areas such as the plantar surface of the foot the role is not so clear.

Melanoma is a rare occurrence before puberty, but shows a gradual increase in incidence from the age of fifteen, peaking at around the age of fifty. Around 80% of lesions occur between the ages of 20-74 years [10]. White populations have a much greater risk of developing the disease than Hispanics, Asians and Afro-Caribbeans. Although non-white races overall have a much lower rate of the disease, they are most likely to develop melanoma in acral locations such as the palmar, plantar surfaces and nail bed [11-15].

Melanoma can arise in a pre-existing naevus (mole) or develop de novo on the skin. The risk of developing melanoma can be correlated to the number of naevi (moles) an individual has. The greater the number-the higher the risk. Dysplastic naevi are atypical moles which are generally larger than ordinary naevi and tend to have an irregular and indistinct border and irregular colours. Patients with dysplastic naevi are also at a greater risk of developing melanoma. Recognised risk factors are listed in Table 1.

**Table 1:** Recognised risk factors for the development of melanoma

General Risk Factors	Risk factors for plantar melanoma*
• Intense and intermittent sunlight and UV radiation exposure • High numbers of benign naevi and dysplastic naevi • Family history of melanoma • A personal history of 3 or more severe sunburns • Immunosuppression (including organ transplant recipients) • Blue or green eye colour • Presence of freckles • Inability to tan • Red hair colour	• High total naevus body counts • Pre-existing naevi on the soles • History of penetrating injury • Exposure to agricultural chemicals

* Based on a single study identifying a number of risk factors for developing plantar melanoma[44].

## Types of melanoma

**Figure 1:**
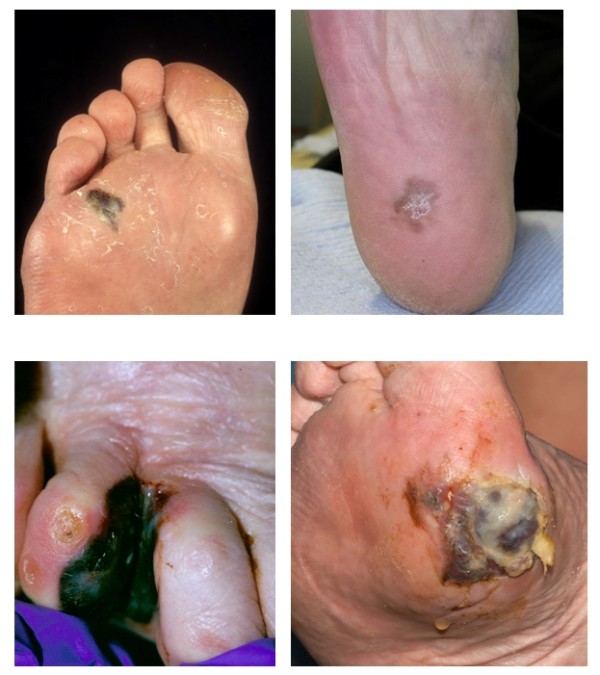
**Various presentations of melanoma on the skin of the foot**.

There are four main types of melanoma although not all can be specifically classified as one particular type (Figure 1).

### Acral lentiginous melanoma (ALM)

This type of melanoma is characterised by having an extensive component running as a layer of malignant melanocytes within the basal layer of the epidermis, giving rise to the term "lentiginous". The term "acral" defines the location which is of the extremities, namely the skin of the hands and feet, including the nail unit. ALM is the only type of MM which arises equally across all skin types and is frequently observed in darker skin types and represents about half of the melanoma occurring on the hands and feet. In the early stages, the clinical symptoms for this type of melanoma maybe very subtle such as an ill defined macule or patch of light brown or grey discolouration of the skin.

### Nodular melanoma (NM)

Nodular melanoma is characterised by a prominent vertical component to the invasion of the tumour when viewed under the microscope. This typically corresponds to a pigmented lesion which may appear nodular to the naked eye. This lesion is more often seen in older patients.

### Superficial spreading melanoma (SSM)

is the most common of the four types so called because of its radial growth phrase (lateral spread) before becoming invasive. It may arise de novo or in a pre-existing mole. This type has been most frequently reported arising on the dorsum of the foot [16].

### Lentigo maligna (LM)

is a type of in situ melanoma, found almost exclusively on the face and neck of older adults in the setting of sun damage. Lentigo maligna may progress to lentigo maligna melanoma which is a lentigo maligna with an area of dermal invasion.

**Figure 2:**
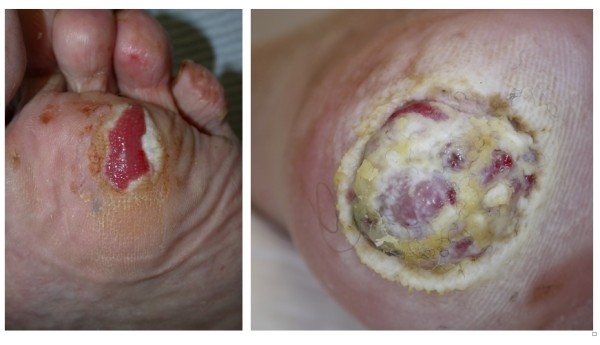
**Amelanotic melanoma arising on the skin of the foot**.

A small but significant proportion of melanoma lack pigmentation and are hence labelled **amelanotic melanoma**. Such lesions are more likely to arise on acral areas such as the feet and be misdiagnosed as other skin disorders as they maybe fleshy in colour (Figure 2).

A large proportion of melanoma are discovered by patients and relatives [17]. Unfortunately, for many patients, the foot is difficult to see and is seldom checked. Consequently, changes may not be readily observed or noted by the patient. Chiropodists/Podiatrists can play an important role in screening the foot and leg.

The prognosis for melanoma corresponds to the histological (Breslow) thickness of the excised tumour. This represents a measure of depth of invasion of the tumour into the dermis. For example, a < 1 mm thick lesion has a five year survival rate of 95%, whilst a > 4 mm thickness holds a 50% chance of survival at five years. As depth of tumour is partly related to its age early identification of suspect lesions is paramount [18].

## Assessment

It is suggested that at an initial appointment details of any pigmented or solitary lesion arising on the feet is recorded in the patient's notes with a description including location, size, colour and shape. Inclusion of accurate measurements can be more objective. The examination must be comprehensive and include interdigital areas and the plantar surface.

When assessing lesions, a history of trauma should not exclude the possibility of a melanoma. Evidence suggests many cases of melanoma are brought to the attention of the patient by co-incidental trauma and injury. The role of trauma in the aetiology of melanoma remains controversial, but it may bring the patient's attention to an existing lesion.

The use of the simple acronym ABCDE [19] is a useful tool in remembering the main clinical signs of a potential melanoma (See Table 2) but may miss amelanotic or smaller lesions [20]. Any mole or solitary vascular lesion whether new or pre-existing which is growing or changing shape or colour should be referred for a specialist opinion.

**Table 2:** The ABCDE acronym

A	Asymmetry. One half of the lesion is not identical to the other.
B	Border. A lesion with an irregular, ragged or indistinct border.

C	Lesion has more than one Colour present within it.

D	Diameter. The lesion has a diameter of greater than 6 mm.

E	Evolution. Any change in the lesion in terms of size, shape or colour.

The utility of the standard ABCDE system for plantar and nail lesions has been questioned owing to the variation in presentation on the plantar surface and within the nail unit compared to other areas of the skin [21-23]. Moreover, data has highlighted how melanoma on the foot holds a poorer prognosis than melanoma elsewhere due to delays in presentation and misdiagnosis of the condition [23-25] particularly so when located in the periungual areas, beneath or around the nails [26]. Lack of pigmentation in suspect pedal lesions can compound the problem. Many misdiagnoses are made in favour of more benign conditions such as:

• Ingrowing toe nail

• Foot ulcer

• Wart/verrucae

• Tinea Pedis/Onychomycosis

• Bruising

• Foreign body

• Sub-ungual haematoma

• Pyogenic granuloma

• Poroma

• Hyperkeratosis-corns/callus

• Necrosis

• Paronychia

• Ganglion

As many of the benign conditions are very common, identifying a rare occurrence of melanoma amongst them can be challenging. In view of the additional difficulties the authors offer an alternative acronym to highlight potential melanoma on the foot using the acronym "CUBED" (Table 3).

**Table 3:** The "CUBED" acronym for foot melanoma

C	**C**oloured lesions where any part is not skin colour.
**U**	**U**ncertain diagnosis. Any lesion that does not have a definite diagnosis

**B**	**B**leeding lesions on the foot or under the nail, whether the bleeding is direct bleeding or oozing of fluid. This includes chronic "granulation tissue".

**E**	**E**nlargement or deterioration of a lesion or ulcer despite therapy

**B**	**D**elay in healing of any lesion beyond 2 months.

Refer when any two features apply.

Clinical judgement should identify lesions which appear "unusual" in their form or have atypical features. For example, the appearance of a suspicious foot ulcer in a patient without the normal risk factors (neuropathy, diabetes etc) should raise concerns as to the correct diagnosis. Furthermore, when individual skin lesions don't respond to a treatment in the normal, timely manner the original diagnosis should be re-considered.

Dermoscopy has been demonstrated to be a useful adjunct in the visual assessment of pigmented lesions to detect potential melanoma on acral skin [27] however, such equipment requires training and knowledge before use. Readers are referred to the article by Bristow and Bowling [28].

## Nail unit melanoma

Like elsewhere on the foot, melanoma of the nail unit (NUM) is typically diagnosed at a later stage in its evolution than melanoma at most other body sites. Accordingly, the tumours are thicker and there is a worse prognosis than for other melanoma. A large UK survey of 4 regions demonstrated that NUM represented 1.4% of melanoma over a 10 year period, giving an incidence of 1 per million of population per year. The 5 year survival of this group was 51%, where those with a Breslow thickness of less than 2.5 mm had a 5 year survival of 88% and those for which the thickness was 2.5 mm or greater, had a 44% 5 year survival rate [29].

## Presentation of melanoma in the nail unit

**Figure 3:**
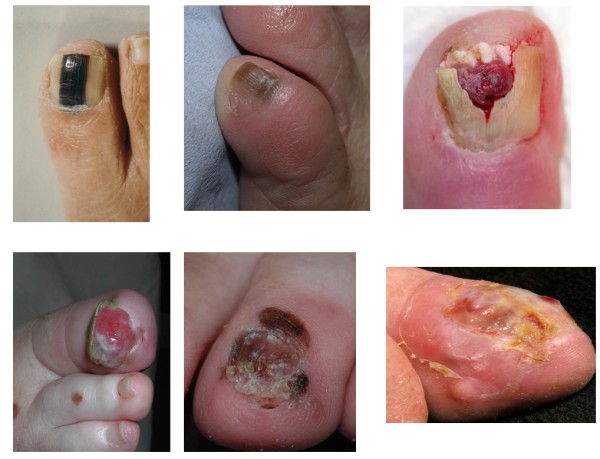
**Various presentations of nail unit melanoma**.

There are 2 main patterns of nail unit melanoma (NUM); longitudinal melanonychia and amelanotic tumours (Figure 3). The first may be associated with alteration of nail plate anatomy in more advanced cases. The latter is almost always associated with nail plate change. Some NUM may present with features common to both patterns.

## Differential diagnosis: Melanoma or haematoma?

**Figure 4:**
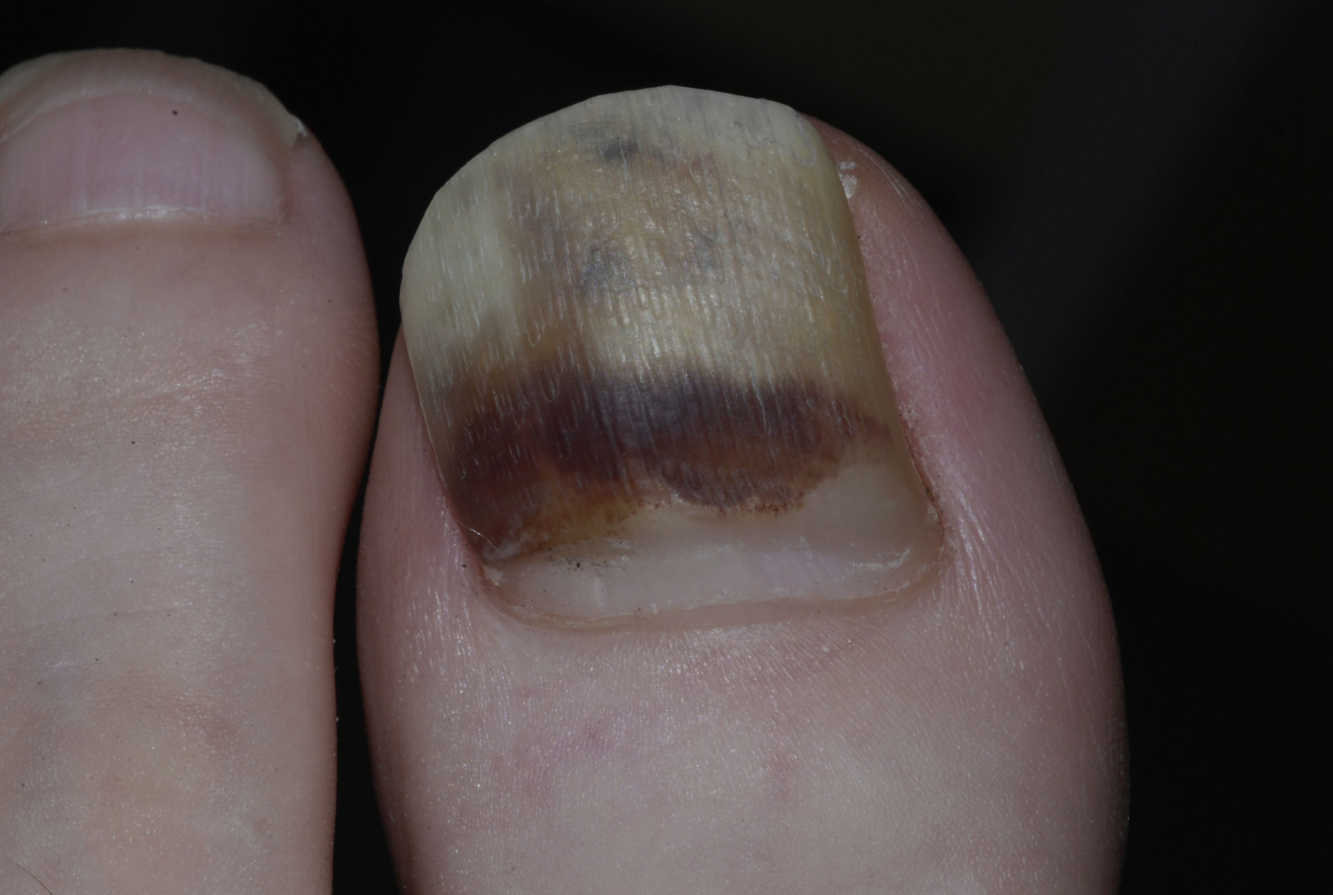
**Subungual haematoma**. Demonstration of haematoma by clear nail growth proximally.

The most common clinical presentation to cause uncertainty is subungual bleeding. The history can be of great value. A subungual bleed will normally have arisen within a day or two and may be associated with an episode of trauma, or more commonly, a period of vigorous activity or sport where no trauma is recollected. Having been noted, it will not change greatly, although the clinician will note a distal drift with time if they review over a period of several months [30] (Figure 4). Associated with this drift a small transverse groove will often emerge from beneath the nail fold about 2 months after the cause of the bleed. This represents a step disturbance of nail plate production, precipitated by the same episode that caused the bleed, but emerging later as it requires the nail to grow by the length of the proximal nail fold before the sign is manifest. Clinical photography is of great value in documenting the exact form and dimensions of pigmented marks within the nail unit. It is best done at the outset, where change over 3 months can provide very useful clues. A source of pigment that clears proximally as it progresses distally will almost always be subungual blood.

**Figure 5:**
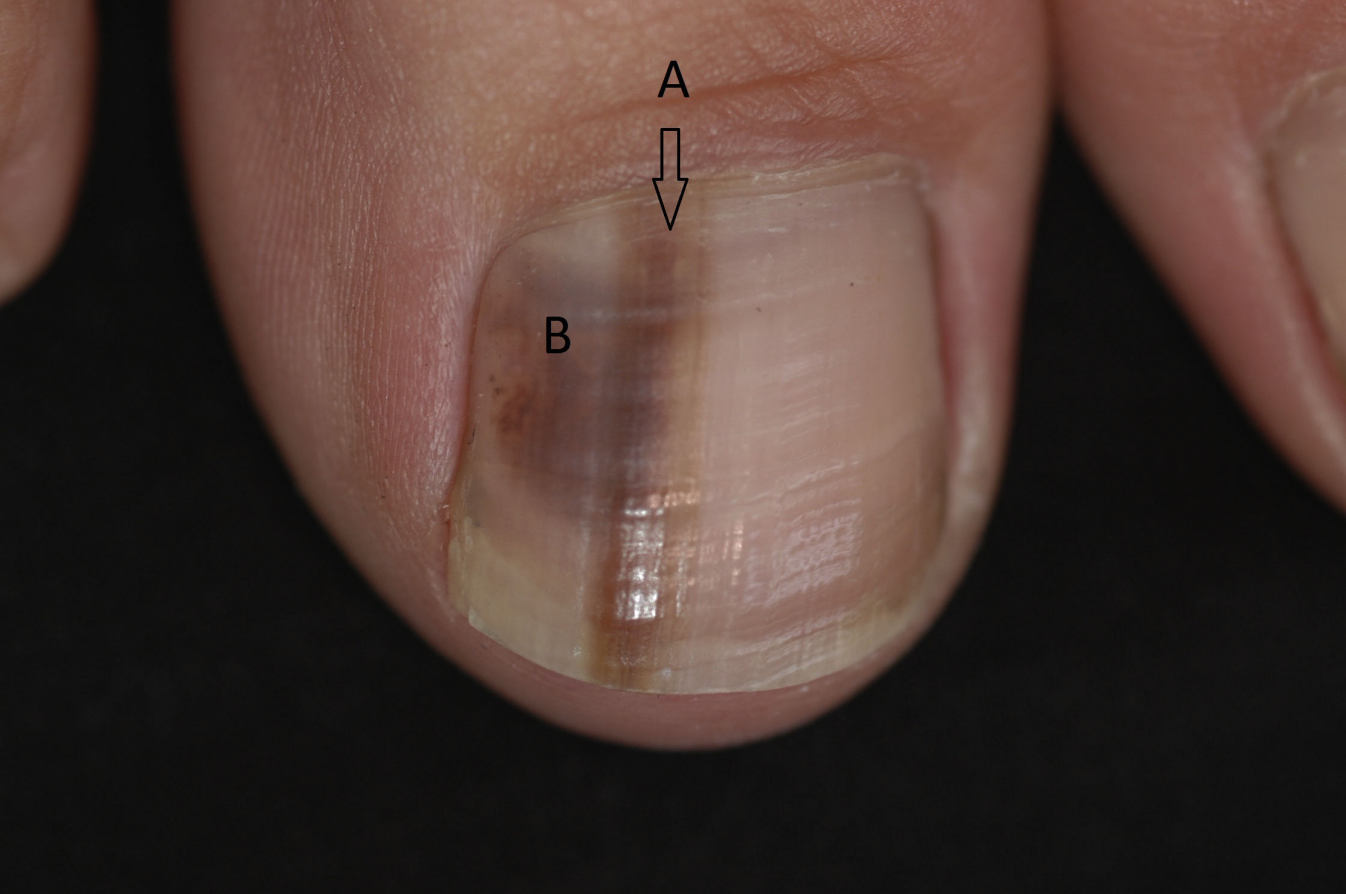
**A single nail exhibiting both longitudinal melanonychia and haematoma**. A: Longitudinal melanonychia arising in the nail matrix from the melanocytes. B: Subungual haematoma limited to the nail bed with poorly defined, rounded borders.

Longitudinal melanonychia reflects melanin pigment created during nail plate generation incorporated within the nail plate as it is formed by the matrix (Figure 5). Subungual bleeding (or subungual haematoma) represents blood beneath the nail, which in some instances may be trapped within pockets of nail plate and be carried with it as the nail grows. Both longitudinal melanonychia and subungual bleeding have a range of benign and malignant causes (see Table 4). Clinically they can be distinguished on a series of points (Table 5), where some of these points can be clarified with dermoscopy. The dermatoscope is a hand held instrument that combines a x10 lens with an internal light source. It can be held directly against the nail plate and periungual skin to examine pigment and other characteristics [31]. When used in combination with clear jelly, a continuous medium is established between the light source and the reflective pigments of the nail plate by avoiding an air interface. This greatly improves the amount of information available to enable the clinician to analyse the source of pigment [32]. There are occasions when a malignancy beneath the nail will bleed such that the presence of blood does not rule out malignancy and associated features need to be considered [30,31]

**Table 4:** Causes of melanonychia compared with those of subungual bleeding

Melanonychia	Subungual bleeding
Benign racial melanonychia	Direct trauma
Laugier Hunziker	Indirect microtrauma-end on repetitive trauma
Inflammation	Haemorrhagic tendency lowering threshold for effects of trauma. eg
• Lichen planus	• warfarin
• Chronic paronychia	• leukaemic
• Trauma/friction	• thrombocytopaenia
• radiation	
Medication e.g.	Subungual tumour
• Minocycline	• squamous cell carcinoma
• Chemotherapy	• wart
• HIV disease or medication	• exostosis
	• melanoma
	• pyogenic granuloma
Addison's disease	
Peutz Jeghers	
Subungual naevus	
Benign melanocyte activation	
Melanoma	
Bowen's disease (in situ squamous cell carcinoma)	
Onychomycosis	

**Table 5:** Features of longitudinal melanonychia compared with those of subungual bleeding-all features are generally true, but there can be individual exceptions

Melanoncyhia	Subungual bleeding
The duration of history is from 3-6 months upwards to 20 years or more	The duration of history is rarely more than 6 months and is typically shorter
A history of trauma is quite common	A history of trauma or precipitating activity is quite common
Lateral margins within the nail are mainly straight and longitudinally oriented	Lateral margins may be irregular
Where margins merges with the nail fold, pigment may spread onto nail fold (Hutchinson's sign)	Pigment rarely extends from beneath the nail plate
There are rarely any detectable transverse features	There may be a proximal transverse groove and/or transverse white mark within the nail
In the absence of clinical tumour, nail plate pigmentation is in continuity with a single zone	Haemorrhage may be broken up into a number of zones
Dermoscopy reveals	Dermoscopy reveals
• continuous pigment between proximal nail fold and distal free edge	• Pigment may not be continuous in the longitudinal axis, with clear nail at either the proximal or distal margin
• in the transverse axis, pigment may vary-whereas in the longitudinal axis it remains largely constant	• Pigment may vary in any axis
• There may be longitudinal flecks of darker pigment within the background pigment of the nail	• Droplets of blood may be seen separated from the main zone of pigmentation
• Pigment is mainly brown black	• Blood may be seen as a discrete layer of material on the lower aspect of the nail plate at the free margin
	• Pigment may be purple black, with increasing red hues at margins. It is rarely brown

One of the biological rules of the nail unit is that functioning melanocytes are limited to the matrix and nail folds, but not found in the nail bed. This means that if pigment change occurs within a structurally normal nail or nail bed, with no continuity with the nail folds or matrix, then it is not likely to be melanocytic and hence cannot be a melanoma. This leads to 2 simple rules:

1. Pigment arising solely within the nail bed with normal matrix and nail folds is not likely to be a melanoma

2. Where melanoma involves the nail bed, there will be a history of the disease starting in the nail matrix or nail fold.

The shape of the outline of the pigmentation is also a useful clue. Blood may present as small irregular pools within the nail bed, with adjacent puddles or drops of purplish brown discoloration. By contrast, longitudinal melanonychia arises as a well organised band of similar width throughout the longitudinal axis, arising in the matrix and extending to the distal edge.

An anecdotal clinical observation is that traumatic causes of subungual bleeding are associated with a proximal white transverse band in many instances [33]. This is more common for trauma to digits of the hand than the foot. The band is likely to represent a physical disturbance to nail production associated with the episode of trauma which in turn will make the nail less translucent for a brief zone. This white band is not seen in melanocytic causes of nail discoloration.

## What is the likely cause of the longitudinal melanonychia?

**Figure 6:**
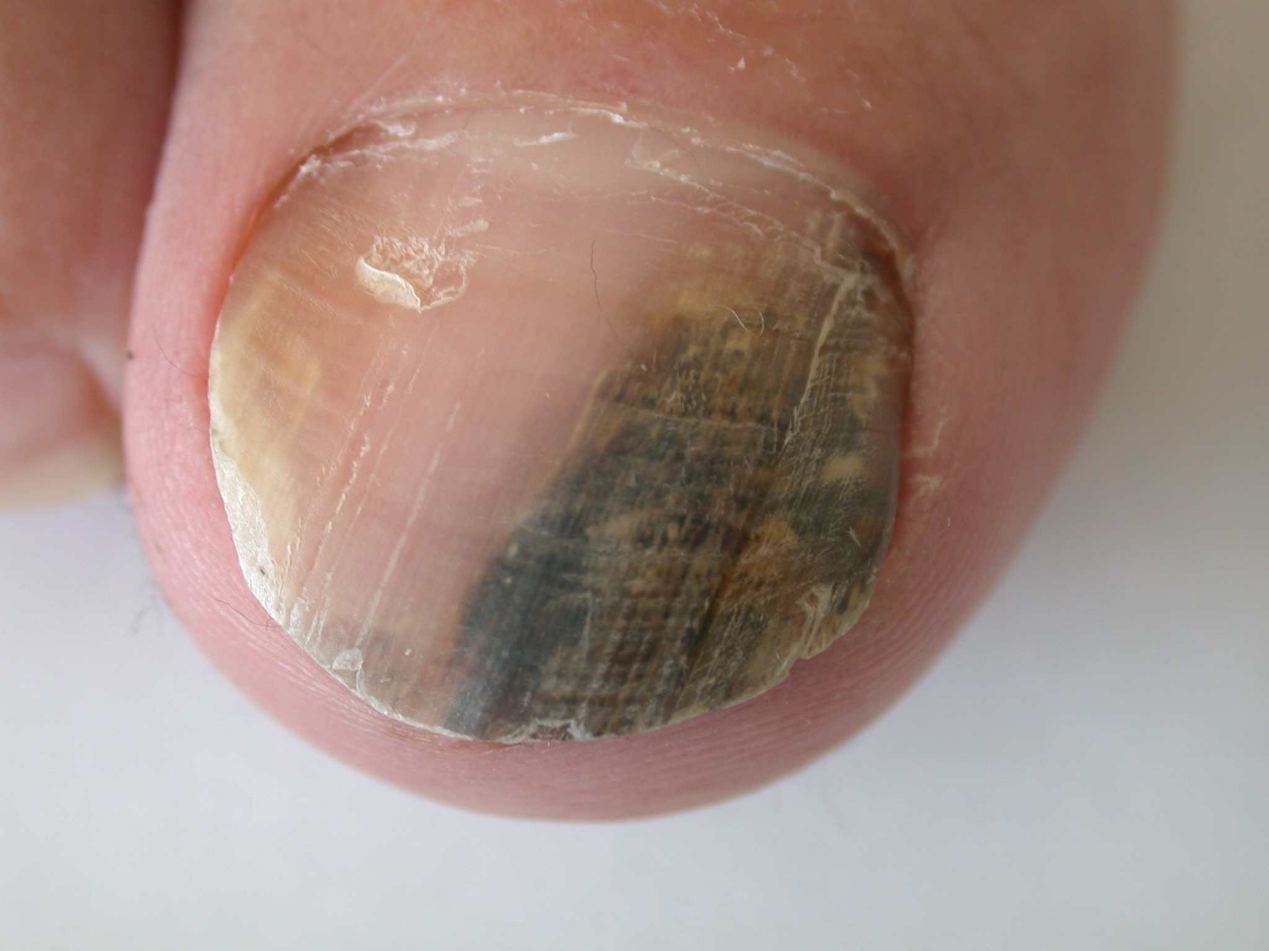
Fungal infection of the nail caused by Fusarium sp. Causing a longitudinal melanonychia

The longitudinal melanonychia most likely to represent malignancy is that arising as a solitary pigmented streak in a white person with fair colouring and of middle age or older. In a dark skinned person, benign nail pigmentation becomes increasingly common with age and is typically found in varying degrees of intensity on several digits. In all instances, there needs to be careful evaluation to determine the cause of the pigmentation [30,34]. If no satisfactory benign explanation can be found, then they should be reviewed by a Dermatologist to consider the need for biopsy. The most common causes are drugs, trauma, fungal infection (Figure 6) and inflammatory diseases such as lichen planus which may be manifest elsewhere on the skin. Both squamous cell carcinoma and melanoma would be considered during assessment. In rare instances, the pigment is exogenous, such as that produced by potassium permanganate. This can be demonstrated by scraping the surface of the nail. Where there is onycholysis, the same may apply to the undersurface of the nail. This is particularly the case where there is colonisation by pseudomonas which can lend a green to black appearance.

**Figure 7:**
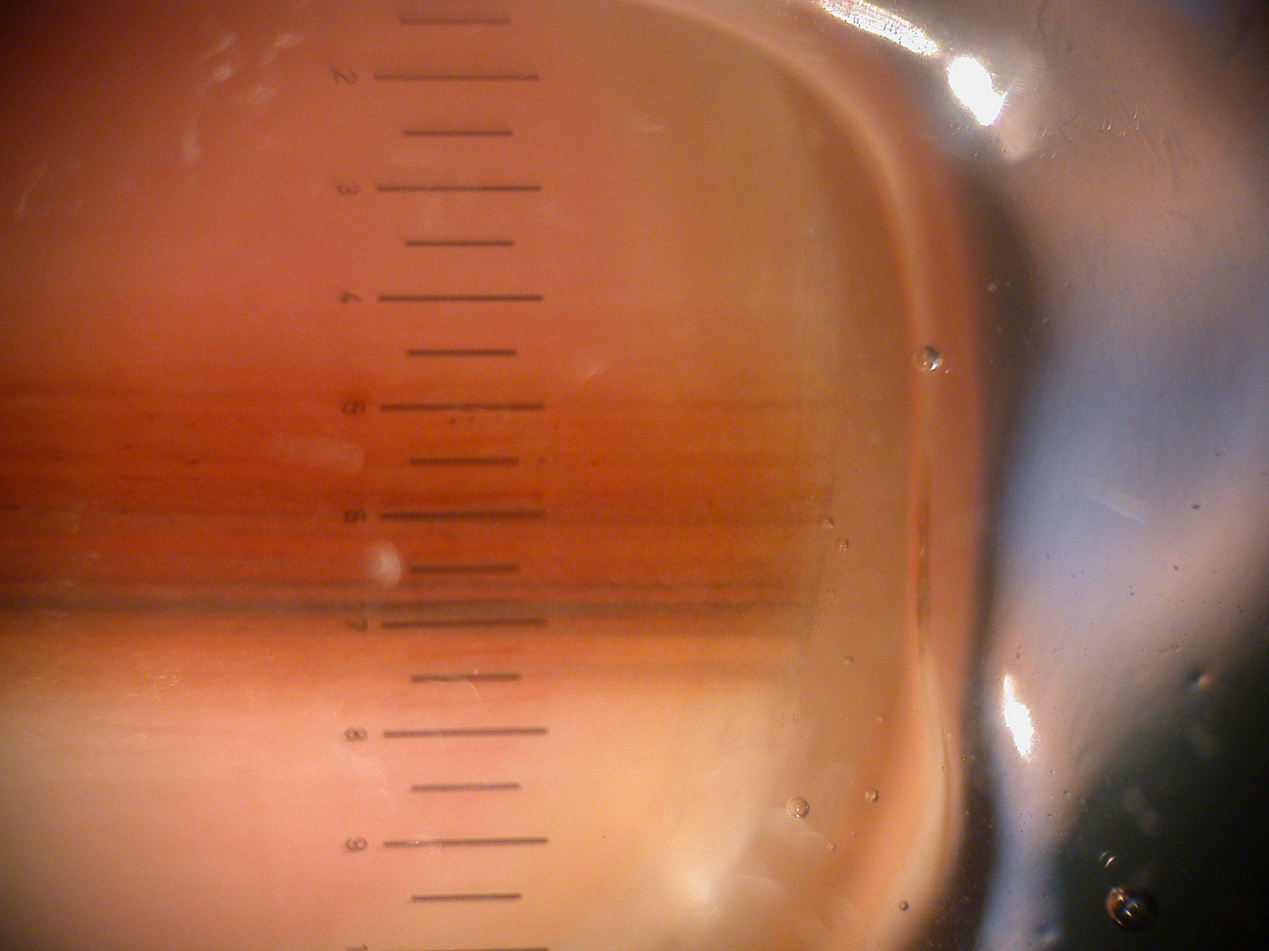
**Dermoscopy of the nail plate demonstrating heterogenous streaks in the longitudinal and horizontal axes**.

Other details for consideration include the pattern of the pigment within the longitudinal streak and whether there is any spread of the pigment onto adjacent skin. Dermoscopy is helpful in both instances and where the pigment is heterogeneous in both the longitudinal and transverse axes (Figure 7), the likelihood of melanoma is greater [31]. Detection of pigment on the nail folds or digit pulp can also be easier with dermoscopy. Where present, it is referred to as Hutchinson's sign after the surgeon of that name noted it in the early historic accounts of subungual melanoma and referred to it as a "melanotic whitlow" conferring a poor prognosis. It is to be distinguished from the "pseudo-Hutchinsons sign" which is the appearance of periungual pigment leant by the melanin within the nail being visible through the translucent edges of the proximal nail fold as it dwindles to a cuticle [35].

Evolution of the pigmentation is diagnostically useful, but not reliable as a means of ensuring that the source of pigment is benign. Whereas blood may be distinguished from melanin over a period of a few months, the characterisation of a benign or malignant source of melanin is less easy. Pigment that does not change is not necessarily benign, however the longitudinal melanonychia that increases in width or variety of pigment is more likely to represent malignancy than one that is static. One exception to this is longitudinal melanonychia in children where the pigment arises in a subungual naevus which changes as the child matures [34]. Quite dramatic nail pigmentation can evolve quickly from a benign lesion and biopsy would rarely be undertaken in this group. A further exception is the evolution of a pigmented streak that comes to be associated with other pigmented streaks on other nails of the hands and feet. This indicates a systemic process and is common in dark skinned races, those taking certain drugs and in a condition termed Laugier Hunziker syndrome. Laugier Hunziker syndrome is increased patchy pigmentation of mucosae of the mouth and/or genitals, associated with multiple homogenous pigmented longitudinal bands in the nails. It is common for this problem to present with one nail in the first instance and hence the value in making a proper examination of all nails and other areas as appropriate [36]. Multiple pigmented bands in dark skinned people may also initially be noted in one nail alone, but are soon detected in others.

## The abnormal nail plate associated with pigment

A nail plate that is structurally altered presents a different scenario. Where there is a longitudinal melanonychia associated with loss of nail integrity this raises concern and needs immediate assessment. In other instances, the pigment may be broken up or scattered within a creamy yellow nail plate. Where there is no preceding history of longitudinal melanonychia, this may represent a pigmented onychomycosis with damage to the nail plate. This can be difficult to assess. Unlike melanocytic pigment which starts in the matrix, the pattern of onychomycosis usually extends from the distal free edge with proximal progression. Early reassurance can be given if the pigmented change and dystrophic nail can all be trimmed away with no disturbance of surrounding skin and there is no sign of a more proximal origin to the pathology. Suspicion of fungus should always be explored by mycological assessment and in particular culture. There is a wide variety of potential organisms [37,38]. Some of the pigmented fungi are non-dermatophytes and may represent a therapeutic challenge likely to be surmounted only if the pathogen is known.

Levit has used a modification of the ABCD rule developed for detection of suspicious pigmented lesions on the skin and applied it to the nail unit [39]. First is A for Age, in the 5^th ^to 7^th ^decade of life. B stands for a Band (longitudinal streak) that is brown or black and measures 3 mm or more. C stands for Change in the nail band or *lack *change in the nail morphology in spite of presumed adequate treatment. D stands for the Digit most commonly involved, which for the foot would be the big toe. E stands for Extension of the pigment onto the adjacent skin or nail fold, known also as Hutchinson's sign and F stands for Family history of melanoma or dysplastic naevus. All these points are reasonable and may guide the practitioner to seek advice (Table 6). They may in turn help the dermatologist when deciding to do a biopsy, although all the other points raised in the preceding text would be considered in taking this step. However, a final diagnosis of melanoma will depend on the histology.

**Table 6:** The ABCDE of nail melanoma after Levit [39]

A	Age Range 20-90, peak 5^th ^-7^th ^decades.
**B**	Band (nail band): Pigment (brown-black). Breadth > 3 mm. Border (irregular/blurred).
**C**	Change: rapid increase in size/growth rate of nail band. Lack of change: failure of nail dystrophy to improve despite adequate treatment.
**D**	Digit Involved: Thumb > hallux > index finger > single digit > multiple digits.
**E**	Extension: Extension of pigment to involve proximal or lateral nail fold (hutchinson's sign) or free edge of nail plate.
**F**	Family or personal history: Of previous melanoma or dysplastic nevus.

## Amelanotic tumour of the nail unit

Amelanotic melanoma arises in the nail unit as it is does at other acral locations, at a rate higher than other body sites. The lack of overt pigment appears to delay the diagnosis further, which in turn affects prognosis [25]. There may sometimes be small pigmented tints to an otherwise pink or granulomatous mass [31]. The differential diagnosis of amelanotic melanoma is considered for all pyogenic granuloma, which is a common benign diagnosis presenting as a vascular nodule. Pyogenic granuloma is usually found on the fingers or toes, bleeds easily and does not readily remit. In Dermatological practice, a pyogenic granuloma would normally be surgically removed. This provides histology to ensure that it was not a melanoma at the same time as resolving the clinical complaint. In biological terms, pyogenic granuloma has much in common with the granulation tissue of ingrowing toenail. Amelanotic melanoma presenting as a granulating mass of the nail fold can be interpreted as an ingrowing nail. This is a well recognised pitfall in podiatry and a potential cause of delayed diagnosis which compromises prognosis [40-43]. Where practice entails cauterising or simply dressing fleshy granulomatous masses of the extremities there is a significant risk of leaving a malignancy undiagnosed. In the authors' experience patients with advanced amelanotic melanoma of the hand or foot often say "they treated it with dressings for the last X months and it just wouldn't heal". Although this article is examining presentation and diagnosis of acral melanoma, squamous cell carcinoma can also present this way and hence the value in asking for histological assessment of any lesion that does not resolve in 2 months, but which oozes or bleeds or has no clear diagnosis. Concern is greatest when the tumour causes disturbance of nail integrity as it arises in the nail matrix and destroys the specialised nail matrix epithelium such that it can not produce nail.

In conclusion, NUM is best detected early if all clinicians and patients have a low threshold for asking for advice early. In particular this means avoiding prolonged periods of conservative management of change in the nail or periungual tissues that are limited to one digit and do not respond promptly to appropriate treatment. For less advanced lesions, where there is only altered pigment, if such pigmentation is limited to a single digit and cannot confidently be attributed to a single episode of subungual bleeding then expert advice should be sought. In all instances, although general practitioners are a good source of general assessment, they typically do not have any experience of NUM. We would recommend assessment by a Dermatologist.

## Referral

If a melanoma is suspected, the normal route for referral would be to a general practitioner. Occasionally, direct referral to the dermatology department may be possible, but local policies will dictate this. Under current NICE guidelines in the UK, patients with suspected melanoma should be seen by a specialist within two weeks of presentation. As a diagnosis of melanoma is relatively uncommon and can only be made after a full professional assessment and biopsy, practitioners should be cautious and not speculative when giving any advice to the patient about potential diagnoses to prevent any unnecessary alarm and concern. A point to emphasise to all patients is that it is important to know the diagnosis of what is being treated. If that diagnosis is not clear, or becomes unclear due to unusual clinical response to development, then both patient and the practitioner need the benefit of a clear diagnosis.

## Summary points

• Melanoma can occur on any part of the foot, including the nail unit, in all ethnic groups and skin types.

• Early recognition and diagnosis can significantly improve prognosis.

• Melanoma of the foot is frequently misdiagnosed, especially when lesions are amelanotic or arise within the nail unit.

• The use of the "ABCDE" and "CUBED" acronyms may improve practitioner's assessment of unusual lesions.

• Any skin or nail lesion arising on the foot with an unclear diagnosis, which deteriorates or fails to heal within two months despite treatment or exhibits unusual features should be reassessed, and referred if considered appropriate..

## Consent

Written informed consent was obtained from the patient for publication of this case report and accompanying images. A copy of the written consent is available for review by the Editor-in-Chief of this journal.

## Competing interests

The authors declare that they have no competing interests

## Authors' contributions

The paper was initially drafted by IB and DB. RT, KA and JB reviewed the manuscript and made suggested amendments. All authors provided images and read and approved the final manuscript.
